# Current Evidence Does Support the Use of KT to Treat Chronic Knee Pain in Short Term: A Systematic Review and Meta-Analysis

**DOI:** 10.1155/2021/5516389

**Published:** 2021-03-23

**Authors:** Wen-hao Luo, Ye Li

**Affiliations:** ^1^Department of General Surgery, Peking Union Medical College Hospital, Chinese Academy of Medical Sciences and Peking Union Medical College, Beijing 100730, China; ^2^Department of Orthopedics Surgery, Peking Union Medical College Hospital, Chinese Academy of Medical Sciences and Peking Union Medical College, Beijing 100730, China

## Abstract

**Objective:**

To demonstrate whether KT is better than placebo taping, nonelastic taping, or no taping in reducing pain.

**Methods:**

PubMed, Embase, Web of Science, the Cochrane Central Library, and ClinicalTrials.gov were systematically searched up to 20 October 2020 for randomized controlled studies that used KT to treat chronic knee pain according to PRISMA guidelines. We extracted the mean differences and SD in pretreatment and posttreatment for selected outcomes measured in the experimental and control groups for subsequent meta-analyses.

**Results:**

In total, 8 studies involving 416 participants fulfilled the inclusion criteria. Our results indicated that KT is better than other tapings (placebo taping or nonelastic taping) in the early four weeks. The mean difference was −1.44 (95% CI: −2.04–−0.84, *I*^2^ = 49%, *P* ≤ 0.01). Treatment methods which were performed for more than six weeks (0.16 (95% CI: −0.35–0.68, *I*^2^ = 0%, *P*=0.53)) show no significant difference in reducing pain. In studies in which visual analogue scale was measured, a positive effect was observed for KT combined with exercise program training (−3.27 (95% CI: −3.69–2.85, *I*^2^ = 0%, *P* < 0.05)).

**Conclusion:**

KT exhibited significant but temporary pain reduction.

## 1. Introduction

Chronic knee pain is an essential cause of pain, disabilities, and reductions in people's quality of life [1]. Chronic knee pain is created by chronic diseases which are commonly knee osteoarthritis (OA) [2], patellofemoral pain syndrome [3, 4], after anterior cruciate ligament reconstruction [5], and pes anserinus tendinitis [6]. Surgery may provide significant therapy effects, but they are not widespread because of the risk of complications in operation and anesthesia. Consequently, the nonoperative measures are an optimal option for most patients with chronic knee pain, such as functional motion, reducing weight, pain killers, and corticosteroid injections. However, current evidence did not prove which one of those nonoperative therapies should be recommended as the first-line therapy based on comprehensive and individualized consideration of the adverse impacts and positive outcomes of patients [7]. Complementary therapy such as ginger in the management of chronic knee pain has been fully discussed in recent studies [8, 9].

Recently, KT has been applied in treating chronic knee pain as a novel nonoperative therapy [10]. Unfortunately, the positive outcome of KT in reducing chronic knee pain is still in dispute [10]. Many studies [11, 12] failed to prove the effectiveness of KT for chronic knee pain in clinical practice. Some studies [13–15] have proved that KT did reduce chronic knee pain, but there is no significant difference compared with other nonoperative therapies. At present, KT is mainly used to improve athletes' short-term muscle pain to improve athletic performance, but the effect of KT on reducing chronic pain caused by some chronic degenerative diseases is uncertain. Admittedly, there was no review of KT for chronic knee pain and no review proved the KT has a superior advantage in pain reduction compared with other approaches. With increasing clinical trials being carried out to evaluate the beneficial effects of KT for chronic knee pain, we grasped this opportunity to conduct a meta-analysis exploring the function of KT. Therefore, the aim of this article was to examine and summarize the evidence of recent RCTs regarding the effectiveness of KT [11–26]. We hypothesized that KT would result in significant pain reduction in early 4 weeks but no significant pain reduction after 4 weeks, which is the focused clinical question in this systematic review. However, there are some uncertainties and conflicts that underlie the hypotheticals. First of all, the relationship between KT application techniques and pain reduction remains controversial. Secondly, whether such pain relief is self-recovery of body function or the true function of KT is still uncertain. Last but not least, does the short-term effect of KT on chronic knee pain patients have its clinical significance? The answer is debatable. It is exceedingly important to figure out those hidden mysteries and help those individuals who believe the long-term benefits of KT get out of their misunderstandings. Therefore, we conducted a systematic review and meta-analysis to test this hypothesis.

## 2. Methods

### 2.1. Search Strategy

Relevant studies were searched and identified by individually searching the following databases: PubMed, Embase, Web of Science, the Cochrane Central Library, and ClinicalTrials.gov up to 20 October 2020 according to PRISMA guidelines. For all databases including grey literatures, reference list of related systematic reviews, and other related studies, the following search strategy was used with database-specific truncation terms: knee AND (tape OR Taping) AND pain (((osteoarthritis) OR (degenerate)) AND ((tape) OR (taping) OR (kinesiotape)) AND ((knee) OR (patellofemoral) OR (tibofemoral)) AND Clinical Trial [ptyp]. Eligibility assessment was performed by LUO Wen-Hao. Disagreements between reviewers were resolved by group discussion and consensus.

### 2.2. Inclusion and Exclusion Criteria

**Figure 1:**
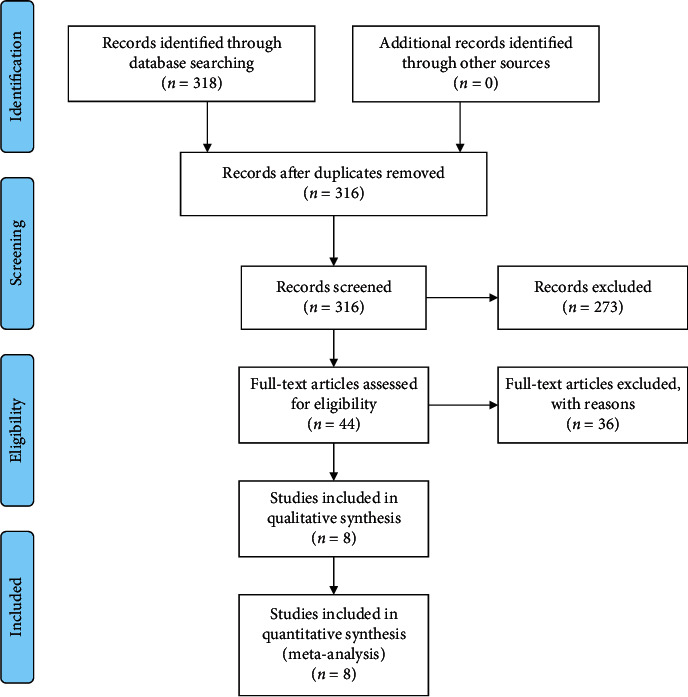
Inclusion and exclusion chart.

The protocol was established a priori. Eligibility was assessed by 2 independent reviewers (LUO Wen-Hao and LI Ye), with the consensus reached by discussing conflicts with a third investigator (Ruoyu Ji). Assessments were performed and repeated twice. Firstly, titles and abstracts were assessed. Then potentially qualified studies were obtained in full text and assessed through the PRISMA flow diagram chart [27] (Figure 1). All authors were familiar with the authorship of studies. There were no restrictions on the history of knee pain, nor on follow-up duration or taping times. In order to evaluate the combined effects of KT with other concurrent interventions, we included those relevant studies. Non-English studies were excluded.

Inclusion criteria are presented in Box 1.

Box 1. Inclusion criteria

Design
    Randomised controlled trials
Publication languages
    English only
Publication year
    Up to October 2020
Patients
    Adults
    People with chronic knee pain diagnosed with knee OA, patellofemoral pain syndrome, pes anserinus tendinitis, or after anterior cruciate ligament reconstruction
Intervention
    KT method
Outcome measure
    Chronic knee pain

### 2.3. Data Collection and Analysis

Data such as authors, publication year, baseline information of participants, dropout rates, assessment time, and the outcome of the KT group and the control group (i.e., placebo taping, no taping, or other intervention) were extracted from each included trial.

### 2.4. Assessment of Study Quality

The methodologic quality of selected studies was blindly evaluated by 2 independent reviewers (LUO Wen-Hao and Li Ye). Disagreements between LUO Wen-Hao and Li Ye were discussed by the group and resolved by a third assessor. Quality was assessed using the Consort 2010 statement as well as Cochrane Library Scale.

### 2.5. Data Synthesis

The primary outcome of this meta-analysis is the comparison of short-term effect and long-term effect of KT in pain reduction. The secondary outcome is the comparison between Kinesio Taping combined with exercise program, and exercise program only comes to the secondary outcome. Pain scores (outcome) were transformed into percentages of the maximum possible score and reported as centimeters on a 10 cm analog scale, using the pain visual analogue scale (VAS), a standardized 11-point scale with a score of 10 being the most painful. The mean difference and standard deviation between KT and other interventions were determined. Effect sizes for KT and other interventions were derived by dividing the mean differences by the pooled SD. Data were entered in Cochrane Collaboration's Review Manager program (RevMan version 5.3, Cochrane Collaboration, Oxford, UK). We analyzed the standardized mean differences and 95% CIs and performed tests of heterogeneity (*I*^2^) for outcomes. Fixed-effects or random-effects models were used appropriately and accordingly. The heterogeneity quantity was used to test heterogeneity between RCTs in our analysis. Moderate-to-high heterogeneity (*I*^2^ = 50%) was explored using sensitivity analyses.

## 3. Results

### 3.1. Study Selection

318 unique articles were selected, of which 8 fulfilled the inclusion criteria (Figure 1). The retrieved data of the 8 eligible studies are listed in Table 1.

### 3.2. Data Analysis

**Figure 2:**
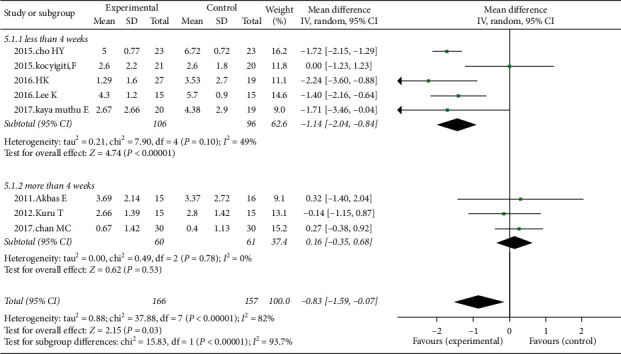
Effects of Kinesio Taping on chronic knee pain. Subgroup analysis: those studies in which the measurement was performed at less than 4 weeks are compared with the remaining 3 studies in which the assessments were performed at the six-week end in pain reduction.

**Figure 3:**
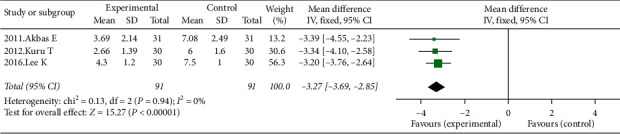
Effects of Kinesio Taping on chronic knee pain. Kinesio Taping combined with exercise program compared with exercise program only.

8 studies investigated the effects of KT in pain reduction. Those studies in which the measurement has been performed for less than 4 weeks (5 studies, 202 participants) showed a mean difference in pain reduction of −1.44 (95% CI: −2.04–−0.84, *I*^2^ = 49%, *P* ≤ 0.01) (Figure 2). 3 studies (2012.Kuru T, 2017.chan MC, 2011.Akbas E, 132 participants) in which the treatment methods have been performed for 6 weeks showed no obvious difference in pain reduction (0.16 (95% CI: −0.35−0.68, I^2^ = 0%, *P*=0.53)) (Figure 2). KT combined with exercise program decreased pain significantly compared with exercise program only (3 studies, 91 participants) (−3.27 cm (95% CI: −3.69– −2.85; *P* ≤ 0.01; *I*^2^ = 0%)) (Figure 3).

## 4. Discussion

This meta-analysis is the first study to put up with the temporary effects of KT on chronic knee pain relief compared with other taping methods. With the current evidences, we suggest KT temporarily controls the symptoms of chronic knee pain and KT should be applied with exercise program in relieving knee pain.

The underlying mechanism of chronic pain reduction in reference to KT remains unknown. The mechanism of its pain relief function is KT can enhance the spaces between skin and muscle by dragging skin, which as a result relieves local pressure and helps to accelerate circulation and promote lymphatic drainage [28]. Therefore, it can reduce pain, attenuate swelling, and alleviate muscle spasm [29]. Moreover, KT increases pressure on the knee joint which may constrict knee joint movement, improve joint stability and functional performance, and thus temporarily minimize the chance of joint injuries.

In terms of the results, we need to clarify the cause of chronic knee pain, whether it is due to aseptic inflammation caused by exercise, trauma, or strain, or OA caused by the wear and regression of articular cartilage. The pain caused by aseptic inflammation can be well alleviated through KT because the inflammation and pain are often transient, so the short-term effect will be better. But for some patients with OA or joint degeneration, KT cannot turn the worn cartilage into a new one, or repair it; therefore, that is why it cannot achieve the purpose of the long-term application. In addition, in terms of comfort and convenience, KT can be used for up to 7 days in chronic exercise pain because 7 days of rehabilitation is sufficient for exercise-induced transient injury. But for chronic knee pain caused by OA with more than one month or two months, KT is not of great significance. KT has been used to improve lymphatic circulation and strengthen joint stability, but it does not eliminate inflammation, nourish cartilage, or even strengthen the surrounding muscles. Therefore, KT is effective for temporary treatment and cannot be used as a long-term treatment.

For the application of KT in patients with OA, a large part of our treatment emphasizes the basic treatment, including weight control and strengthening of the surrounding muscles. During our basic treatment, some patients feel knee pain because of inappropriate exercise or excessive exercise. Then we can use KT to restrict the excessive exercise of the knee. Its benefits will be more obvious than nonsteroidal anti-inflammatory drugs, with smaller side effects. However, for some people with moderate or severe knee degeneration, the effect of KT will not be very good because KT cannot save cartilage wear back.

Overall, KT is a relatively cost-effective treatment intervention for chronic knee pain. It is commonly used as a sports and rehabilitation programme. However, current evidences from clinical trials regarding pain outcomes are controversial and insufficient to draw conclusions on the effects of KT.

Some limitations should be addressed. Firstly, some control groups might expose significant effects to relieve the symptoms for patients with chronic knee pain, which cannot reflect the virtual effect of the KT group when compared with those control groups. Secondly, some studies were of insufficient quality to warrant data extraction to contrast. Thirdly, we might have excluded relevant but non-English studies. Fourth, significant heterogeneity was encountered perhaps due to various regimens, doses, duration, center settings, populations enrolled, etc., calling for cautious interpretation of the results. Fifth, many of the studies suffer from significant sources of bias. Sixth, the effect in many occasions was assessed by very few studies; thus, the evidence to support it is low. Finally, the PROSPRO registration code was not provided.

Remarkably, it should be noted that the application of KT was initially designed for patients with chronic knee pain in the early stage [30]. But we found that the long-term effects of Kinesio taping are not significant and outstanding.

## 5. Conclusion

KT is essential to relieve chronic knee pain and prevent massive use injuries in patients with chronic knee pain but not in a long-term effect. Therefore, KT could be temporarily used in practice for exercise or rehabilitation training.

## Figures and Tables

**Table 1:** Description and baseline information of included studies.

Study (ref.)	Trial design	Diagnosis	Consort 2010 statement	Cochrane Library score	Participants	Dropout rates	Kinesio Taping group	Control group	Assessment schedule	Assessment timing	Outcome measurement	Pain outcome	Data collection
Aydogdu [16], 2017	RCT	Knee osteoarthritis	23	14	*N* = 54 M/F: 8/48	None	Kinesio Taping with Y-shaped and conventional treatment *N* = 28 Age: 52.53 (9.68) years BMI: 31.18(5.14)	Control group: the same conventional treatment alone. *N* = 26 Age: 52.53 (9.68) years BMI: 31.18 (5.14)	Five times per weeks for three weeks	1 hour later after taping in first session, three weeks later	(1) Pain level (2) Range of motion (3) Muscle strength (4) Functional status	VAS	(VAS) experimental: pre: 4.38 ± 1.48; post: 3.37 ± 1.59; p = 0.002 Control: pre: 4.87 ± 1.49; post: 2.76 ± 1.76; *P *≦ 0.01 *P* (post ex vs. control) = 0.070
Kaya [11], 2017	RCT	Knee osteoarthritis	26	22	*N* = 39 M/F: 6/33 Age: 55.58 (6.23) years	Kinesio Taping: 1/21 PT: 2/21	Kinesio Taping group: Kinesio Taping with Y-shaped. *N* = 20. Age: 54.25 (6.01) years M/F: 4/16 BMI: 30.72 (3.80)	Placebo Kinesio Taping group: Kinesio Taping applied transverse to the muscle groups. *N* = 19. Age: 57.10 (6.26) years M/F: 2/17 BMI: 31.34 (6.16)	3 applications with a 3- to 4-day interval between each application	Baseline, after the initial Kinesio Taping, The third Kinesio Taping, 1 month later (follow-up)	(1) Pain level (2) Active ROM (3) Functional status (4) Muscle strength	VAS	(VAS) experimental: baseline: 6.08 ± 3.50; post: 2.67 ± 2.66; score change: 3.41(1.75,5.07) Control: baseline: 6.76 ± 1.92; post: 4.38 ± 2.90; score change: 2.38(1.45,3.30); score change: −1.76(−3.07, −0.45); *P *< 0.05
Wageck et al. [17], 2016	RCT	Knee osteoarthritis	27	19	*N* = 39	Kinesio Taping: 19/38 ST: 18/38	Kinesio Taping group: three simultaneous Kinesio Taping applications *N* = 19 Age: 69.6 (6.9) years M/F: 3/35∗ BMI: 30.0 (4.9)	Sham taping: Sham Kinesio Taping applied transverse to the muscle groups *N* = 20 Age: 68.6 (6.3)years M/F7/31∗ BMI: 31.3 (4.1)	Taping in situ for 4 d	Baseline, Day 4 (end of the taping period), Day 19 (follow-up	(1) Pressure pain threshold (2) Isokinetic muscle strength (3) Physical function (4) Lower-limb swelling (5) Knee-related health status	Pressure pain threshold (PPT) via digital pressure algometry (kgf/cm^2^)	
Lee et al. [19], 2016	RCT	Knee arthritis	12	10	*N* = 30	None	Kinesio Taping group: Kinesio Taping with Y-shaped and general physical therapy *N* = 15 Age: 72.0(4.0) years Height: 160.7(7.1) cm Weight: 64.9(8.8) kg	Control group: conservative treatment and the same general physical therapy *N* = 15 Age: 73.1 (5.8) years Height: 156.3(7.7) cm Weight: 61.1(10.7) kg	Three times per week for four weeks	Baseline, three weeks later	(1) Pain (2) ROM (3) Functional evaluation	VAS	(VAS) experimental: pre: 7.5 ± 1.0; post: 4.3 ± 1.2 Control: pre: 7.1 ± 1.1; post: 5.7 ± 0.9; *P* ≦ 0.01
Kocyigit et al. [14], 2015	RCT	Knee osteoarthritis	24	23	*N* = 41 M/F = 13/28 Age: 45 (15) years	Kinesio Taping: 1/22 ST: 1/21	Kinesio Taping group: Kinesio Taping with Y-shaped and I-shaped *N* = 21 Age: 52(7.5) years	Sham taping group: 5 cm beta fix surgical hypoallergenic flexible tape without tension. *N* = 20 Age: 52(10)years	12-day period	Baseline, the end of taping period (12th day)	(1) Pain level (2) Function (3) The quality of life (via NPH)	VAS	(VAS) experimental: baseline: 5.4 ± 2.1; post: 4.5 ± 2.1; *p* = 0.015 control: baseline: 5.1 ± 1.9; post: 3.7 ± 2.3; *p* = 0.011; *P*=0.242
Kuru et al. [20], 2012	RCT	Patellofemoral pain syndrome (PFPS)	21	15	*N* = 30 Age: 32.9 (12.2) years	None	Kinesio Taping group: Kinesio Taping and an exercise program *N* = 15 Age: 32.93(12.17) years M/F: 3/12 BMI: 23.65(4.59)	The ES group: receiving electrical stimulation and the same exercise program *N* = 15 Age: 40.93(10.57)years M/F: 1/14. BMI: 26.80(3.67)	3 times a week for 6 weeks	At the start of the treatments and at the six-week end	(1) Pain (2) Range of motion (3) Muscle strength (4) Functional condition (5) Quality of life (SF-36)	VAS	(VAS) experimental: baseline: 6.00 ± 1.60; post: 2.66 ± 1.39; *P* ≦ 0.01 Control: baseline: 6.73 ± 1.53; post: 2.80 ± 1.42; *P* ≦ 0.01
Cho et al. [12], 2015	RCT	Knee osteoarthritis	24	20	*N* = 46 Age: 57.9 (4.4) years	None	Kinesio Taping group: Kinesio Taping with Y- or I-shaped *N* = 23 Age: 58.2 (4.5) M/F: 6/17 Height: 161.8 (8.2)cm Weight: 65.7 (8.7)kg	Placebo-Kinesio Taping group: Kinesio Taping without tension or stretch to the rectus femoris in the same manner *N* = 23 Age: 57.5(4.4). M/F: 7/16. Height: 163.3 (7.3)cm. Weight: 68.6 (10.0)kg		Before experiment and 1 hr after taping	(1) Pain (2) Pain-free AROM (3) Proprioception	VAS at rest and during walking, pressure pain thresholds (PPTs)	(VAS)experimental: pre: 6.72 ± 0.91; post: 5.0 ± 0.77; *P* ≦ 0.01 Control: pre: 6.84 ± 0.71; post: 6.72 ± 0.72; *p* = 0.064 *p* = 0.628 *P* ≦ 0.01
Chan et al. [13], 2017	RCT	After anterior cruciate ligament reconstruction	26	19	*N* = 60	Kinesio Taping: 5/35 Con: 3/33	Kinesio Taping group: receiving Kinesiology Taping and the standardized physiotherapy rehabilitation *N* = 30 Age: 27.4 (8.25) M/F: 22/8	Control group: not receiving any form of taping but undergoing the same standardized physiotherapy rehabilitation not receiving any form of taping *N* = 30 Age: 26.3(7.04) M/F: 24/6	At the first and second weekly physiotherapy sessions after the ACLR, each application lasting 5 days (five times per week for 2 weeks)	At the first, second and sixth week	(1) Pain (2) Total range of motion (ROM) of the knee (3) Self-reported knee function (4) Midpatella circumferential girth	VAS	(VAS) experimental: pre: 2.90 ± 1.83; post: 0.67 ± 1.42 Control: pre: 2.18 ± 1.95; post: 0.40 ± 1.13
Homayouni et al. [18], 2016	RCT	Pes anserinus tendino-bursitis	27	15	*N* = 46 Age: 49.9 (6.7) years	Kinesio Taping: 1/28 Con:19/28	Kinesio Taping group: with space-correction technique *N* = 27 Age: 49.85(6.8)years M/F: 4/23 BMI: 28.32(1.7)	Naproxen + physical therapy group: naproxen (250 mg TID for 10 days) and 10 sessions Of daily physical therapy *N* = 19 Age: 50.0(6.6)years M/F: 2/17 BMI: 27.94 .6	Kinesio Taping: applied for 3 weeks, three times with a 1-week interval Con: 250 mg TID and daily physical therapy for 10 days	The beginning and end of the study	(1) .Pain, (2) Swelling scores	VAS	(VAS) experimental: base: 8.07 ± 2.4; post: 1.29 ± 1.6 Control: base: 6.63 ± 3.0; post: 3.53 ± 2.7
Akbas et al. [15], 2011	RCT	Patellofemoral pain syndrome	26	19	*N* = 31, all females Age: 44.88 years		Kinesio Taping group: Kinesio Taping and muscle strengthening and soft tissue stretching exercises *N* = 15 Age: 41.00 (11.26) years BMI:: 25.17 ± 4.80	Control group: the same muscle strengthening and soft tissue stretching exercises *N* = 16 Age: 44.88 (7.75) years BMI: 28.64 (5.77)	Kinesio Taping: five days intervals for six weeks, additionally exercises for six weeks Con: exercises for six weeks	At the first, third, and sixth weeks	(1) Pain (2) Tension of the iliotibial band/tensor fascia lata and hamstring muscles and the mediolateral location of the patella (3) Functional performance	VAS	(VAS)experimental: base: 7.08 ± 2.49; post: 3.69 ± 2.14 Control: base: 6.11 ± 2.43; post: 3.37 ± 2.72

## Data Availability

No data were used to support this study.
